# The holistic model of leukaemia survivorship care: derived from a qualitative exploration of leukaemia survivorship

**DOI:** 10.1007/s00520-025-09382-0

**Published:** 2025-03-28

**Authors:** S. Kirsten, R. Laidsaar-Powell, J. M. Shaw, H. M. Dhillon

**Affiliations:** Psycho-Oncology Cooperative Research Group, Faculty of Science, School of Psychology, The University of Sydney, Sydney, NSW 2006 Australia

**Keywords:** Leukaemia, Survivorship, Model, Qualitative

## Abstract

**Purpose:**

Increasing survival rates have left many leukaemia survivors with debilitating effects from the disease and its treatments. However, little is known about the persistent unmet needs of people living with leukaemia.

**Methods:**

We aimed to qualitatively explore the experiences of individuals living with leukaemia and suitability of the Clinical Oncology Society of Australia’s (COSA) Model of Survivorship Care (2016) to reflect leukaemia survivorship. We used an inductive qualitative approach, conducting semi-structured interviews with leukaemia survivors recruited via social media and cancer advocacy organisations. Interviews were continued until information power was deemed appropriate. Reflexive thematic analysis (RTA) was used to describe and interpret key themes and meta-themes in the data.

**Results:**

Overall findings were examined alongside the COSA Model. Twenty-four leukaemia survivors were interviewed; six themes were identified: (1) leukaemia is impactful, life-altering, and unexpected; (2) leukaemia is enduring, life-limiting, and uncertain; (3) survivorship is a team effort; (4) centrality of work as identity, focus, and financial security; (5) the dynamic landscape of coping; and (6) survivorship as adjusting. Overall, participants described leukaemia survivorship as (1) recursive and (2) holistic.

**Conclusions:**

Our findings, while broadly corresponding with the COSA Model, demonstrate it lacks nuances specific to leukaemia survivorship. We recommended the HMLS be used to guide future leukaemia-specific development of the COSA Model and survivorship services.

**Implications for cancer survivors:**

We identified key domains and stages common across leukaemia survivorship, presented in our proposed Holistic Model of Leukaemia Survivorship (HMLS), addressing these domains are critical to the provision of quality survivorship care.

**Supplementary information:**

The online version contains supplementary material available at 10.1007/s00520-025-09382-0.

Leukaemia is a complex, life-threatening, heterogeneous group of blood cancers. In 2020, leukaemia was the second most diagnosed haematological cancer worldwide with approximately 474,519 new cases and 311,594 deaths [1]. Modern treatments have increased survivorship across all haematological cancers but have resulted in increasing adverse long-term physical, psychosocial, and lifestyle outcomes [2, 3]. While views of survivorship as beginning at diagnosis have been widely accepted, implementation often addresses follow-up care exclusively [4–6]. Despite the need for holistic follow-up care, little is known about the needs of leukaemia survivors across the whole disease trajectory [3, 7, 8].

Research investigating patient experiences across the leukaemia trajectory is limited [8]. Previous research reports significantly compromised health-related quality of life (HRQoL) in people with leukaemia [9–12]. Systematic reviews and qualitative studies have explored haematological cancers collectively and identified fear of cancer recurrence (FCR), need for greater psychological support, and limited access to tailored medical information as prevalent concerns [2, 3, 13, 14].

Diagnosis is a distressing transition into survivorship for many leukaemia patients [15, 16]. Research indicates haematological cancer survivors have difficulty transitioning from in-patient care, adjusting to usual life, managing anxiety and FCR, and navigating physiological side-effects [17–19]. Financial strain is a marked concern, with survivors expressing difficulty with medical expenses exacerbated by inability to work [20].

Cancer survivorship care is complex, involving disease monitoring and secondary disease prevention, alongside physical, psychosocial, and lifestyle interventions [21]. The goal of survivorship care models is to plan and organise delivery of supportive care and is broadly categorised as shared care, specialist-led, primary care provider-led or nurse-led [7]. Current survivorship care models typically implement a one-size-fits-all approach and overlook haematological cancers, which may have different trajectories to solid tumours, omitting the needs of people requiring long-term management [7, 13, 22, 23]. Survivorship models often lack evidence-based guidelines, are established on narrow definitions of survivorship, and not routinely implemented [5, 7, 22, 23]. To facilitate holistic leukaemia survivorship care, patient experiences need grounding in empirically founded models of survivorship.

To our knowledge, no specific survivorship care models have been proposed for haematological cancer survivorship. The Clinical Oncology Society of Australia (COSA) Model of Suvivorship Care (2016) provides a promising theoretical grounding for leukaemia survivorship, on the basis that it: (1) conceptualises survivorship as a recursive continuum starting at diagnosis, warranting tailored intervention at each transition point; (2) delineates survivorship according to diagnosis, primary treatment completion, follow-up care, and time of new problem; and (3) level of care is determined according to ongoing needs assessment during diagnosis and treatment completion, followed by risk-stratification to determine follow-up care.

We aimed to qualitatively explore and conceptualise the survivorship needs of leukaemia patients. Moreover, we aimed to investigate whether the COSA Model (2016) applies to leukaemia patient experiences. This study is exploratory and hypothesis-generating. Therefore, no a priori assumptions were made regarding patient experiences.

## Methods

### Design

This study implemented a cross-sectional exploratory design, using reflexive thematic analysis (RTA) to explore leukaemia survivorship experiences [24]. Qualitative data were collected using semi-structured interviews. This study was approved by The University of Sydney Human Research Ethics Committee (2022/225).

#### Population and sample size

Participants living with leukaemia were eligible if they (a) were aged over 18 years; (b) received a diagnosis of leukaemia as an adult in the past 10 years; and (c) were at least 6 months post-initial and active treatment completion or for those receiving maintenance therapy, at least 12 months post-diagnosis. All participants provided informed consent online. A sample of (*N* = 24) participants was recruited and deemed sufficient according to information power assessment [25].

### Procedure

Participants were recruited through the Leukaemia Foundation Australia, Cancer Council NSW, Twitter, and Facebook haematological cancer groups. Prospective participants used a weblink to access the participant information statement, consent form, and online survey on the REDCap (Research Electronic Data Capture) platform [26]. Consenting participants completed the survey, after which they were contacted to arrange an interview. Interviews were conducted over the telephone and audio-recorded. All interviews were transcribed verbatim using Trint audio transcription software [27]. Data collection continued until the quality of information was determined appropriate with iterative assessment of information power by the research team.

### Materials and measures

#### Demographic information and clinical characteristics

Participants completed a 9-item demographic questionnaire followed by a 5-item clinical characteristics questionnaire assessing type and date of diagnosis, treatments, treatment status (i.e. ongoing), and other chronic health conditions.

#### Interview schedule

Using semi-structured interviews, we explored the following: (1) participant experiences of diagnosis, treatment, and monitoring; (2) physiological and psychological experiences of living with leukaemia; (3) occupational and financial impacts of leukaemia; (4) patient support networks and coping strategies; and (5) patient experiences of follow-up care services. The interview schedule was iteratively revised to maximise question relevance (Supplementary File 1).

### Data analysis

Demographic and clinical characteristics were analysed descriptively in Excel [26, 28]. Semi-structured interviews were analysed in Excel using RTA [29]. RTA entails the following iterative and recursive 6-phase analytical process: (1) familiarising yourself with the data set; (2) coding; (3) generating initial themes; (4) developing and reviewing themes; (5) refining, defining, and naming themes; and (6) writing up [24, 30] (see Supplementary File 2 for detailed procedure).

### Methodological rigour

Several strategies were implemented to ensure methodological rigour, mitigate researcher bias, and ensure trustworthiness. Rigour was assessed using the 32-item Consolidated Criteria for Reporting Qualitative Research (COREQ) [31] checklist (Supplementary File 3) and Braun and Clarkes’ (2023a) recommendations for RTA best practice.

## Results

**Fig. 1:**
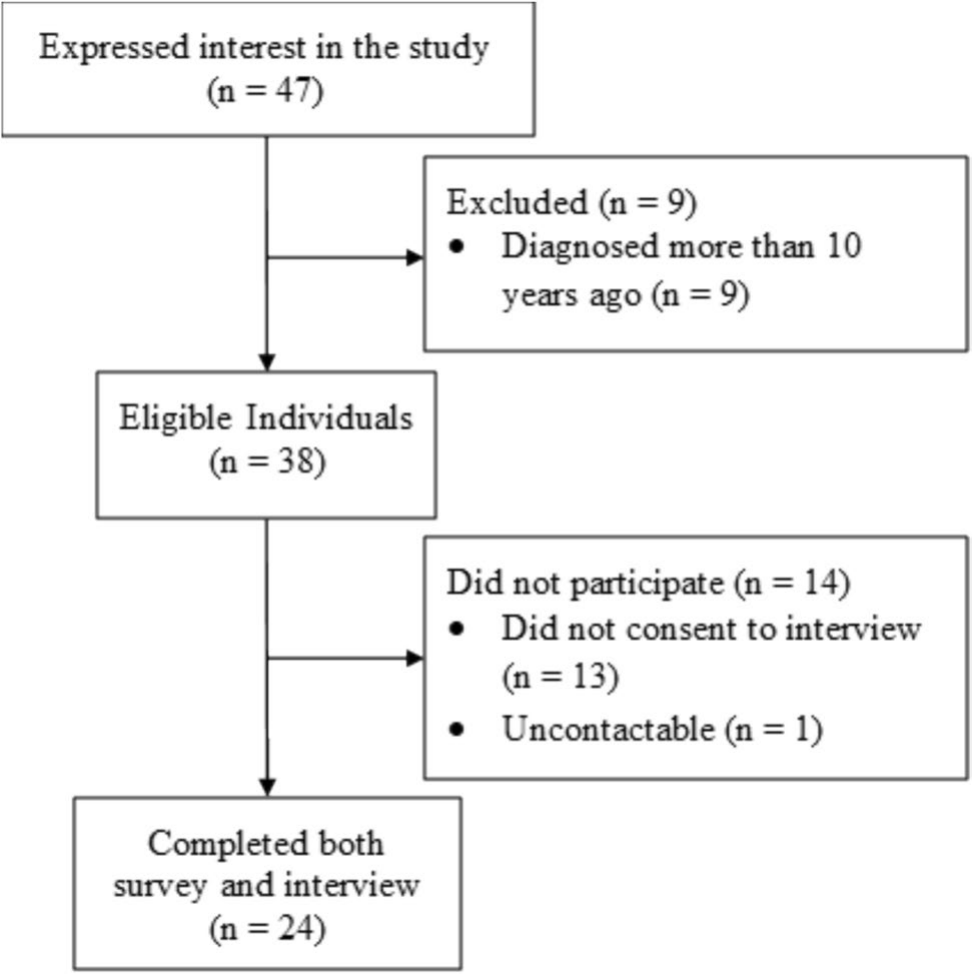
Study consort diagram depicting participant flow through the study

Forty-seven leukaemia survivors consented to participate and completed the online survey. Nine were ineligible, twenty-four completed semi-structured interviews (51% response rate). Median interview duration was 61.5 min (range 40–84); see Fig. 1 for participant flow.

### Sample characteristics

Participants had a median age of 62 years (range 22–80), most were male (63%), married (88%), and originated from three Australian states and one territory (New South Wales = 10; Victoria = 7; Queensland = 5; Western Australia = 1; Australian Capital Territory = 1) (see Table 1 for demographics). Sixteen participants had been diagnosed with heterogeneous subtypes of leukaemia. The median time since diagnosis was 4 years (range 1–9). Eight participants were receiving maintenance therapy.

**Table 1:** Demographic information by participant

Study ID	Age (years)	Sex	Education^a^	Employment^b^ (pre diagnosis)	Employment^b^ (post diagnosis)	Country of birth^c^	Marital status^d^	Other health conditions (chronic or treatment toxicity)?^e^	Leukaemia subtype^f^	Treatment^g^	Ongoing treatment?	Time since diagnosis (year)
P15	55	F	PG	FT	U	A	M	N	ALL	C, BMT, I	Y	2.4
P18	68	F	U	C	R	A	M	Y	AML	C, RT, SCT	N	4
P19	70	F	U	R	R	O	M	Y	CML	T	Y	3.3
P23	30	M	PG	FT	FT	O	M	N	LGLL	I	Y	4.2
P28	57	M	U	FT	FT	O	M	Y	CLL	C, I	N	6.6
P29	22	F	L	FT-S	PT–S	A	S	Y	AML	C, SCT	N	4
P31	42	M	T	FT	PT	A	S	Y	CML	C, T	Y	7.6
P39	68	M	T	FT	FT	A	D	N	AML	C	N	8.1
P56	69	F	PG	C	R	O	M	Y	AML	C, T	N	1.9
P57	44	M	T	FT	FT	A	M	N	BAL	C, RT, BMT, I	N	4.6
P61	31	F	PG	FT	FT	A	M	N	AML	C, RT, SCT	N	2.4
P64	65	M	U	R	R	A	M	Y	BAL	C, RT	N	8.3
P67	22	F	U	FT, FT-S	PT, PT–S	A	M	Y	APML	C, O	N	1.7
P76	68	M	L	FT	PT	A	M	N	CLL	C	N	8.4
P83	80	M	U	R	R	A	M	Y	AML	C	N	9.8
P91	55	F	T	FT	U	A	M	N	AML	C, O	N	3.8
P94	59	M	PG	FT	PT	A	M	N	APML	C	N	1.8
P112	57	M	T	FT	FT	A	M	N	CLL	C, I	N	1.4
P122	72	M	T	R	R	A	M	N	AML	C	Y	1.8
P123	69	M	PG	R	R	A	M	Y	AML	C, RT, BMT	Y	4.3
P126	67	F	PG	FT	R	A	M	Y	AML	C, BMT	N	4.4
P137	50	M	I	FT	FT	O	M	N	CML	T	Y	2
P150	73	M	U	R	R	A	M	N	CLL	C	Y	1.7
P152	71	M	U	C	C	A	M	Y	AML	C, SCT	N	5

^a^Highest level of education attained; I = year 10 or below (intermediate), L = year 12/HSC (leaving), T = TAFE certificate/diploma, U = university degree, PG = higher degree (postgraduate)

^b^Current employment status; FT = full-time employment, PT = part-time employment, C = casual, U = unemployed, R = retired, FT-S = full-time study, PT–S = part-time study

^c^Country of birth; A = Australia, O = other (UK, South Africa, Italy, New Zealand)

^d^Marital status; M = married/de facto, S = single, D = separated/divorced

^e^Whether the participant has other chronic health conditions; Y = yes, N = no; treatment toxicities included graft versus host disease (GvHD), chronic obstructive pulmonary disease, hypothyroidism, irritable bowel syndrome, osteonecrosis, osteoporosis, osteoarthritis, syndrome of inappropriate antidiuretic hormone ADH release, high cholesterol, mental health issues, pernicious anaemia, chronic pain, chronic fatigue, immunodeficiency, diabetes, and kidney disease; chronic health conditions included asthma and lung cancer

^f^Type of leukaemia; AML = acute myeloid leukaemia, APML = acute promyelocytic leukaemia, ALL = acute lymphocytic leukaemia, BAL = bi-phenotypic acute leukaemia, CML = chronic myeloid leukaemia, CLL = chronic lymphocytic leukaemia, LGLL = large granular lymphocytic leukaemia

^g^Initial treatment received for cancer; C = chemotherapy, RT = radiation therapy, SCT = stem cell transplant, BMT = bone marrow transplant, I = immunotherapy, T = targeted therapy, O = other anticancer treatment

### Qualitative findings

**Fig. 2:**
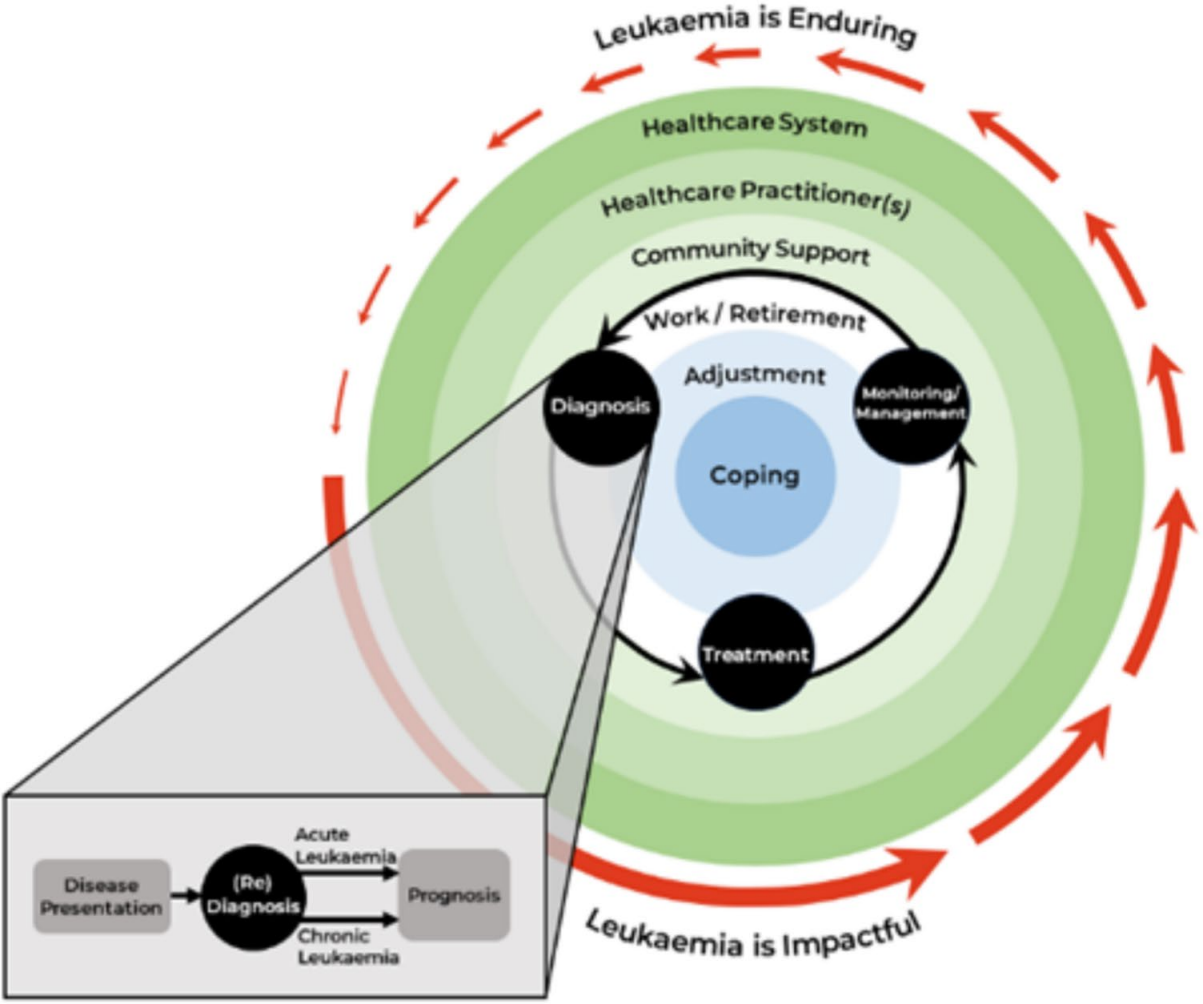
Holistic Model of Leukaemia Survivorship. The HMLS details leukaemia survivors’ recursive transition through medical stages (i.e. diagnosis, treatment, and monitoring/management) (black arrows). Each medical stage is characterised by different biopsychosocial challenges; this is further impacted by leukaemia subtype and personalised risk-stratification. Diagnosis is expanded (grey box) to indicate initial disease presentation, including subsequent re-diagnosis and updated prognosis. The green, white, and blue circles capture the key domains implicated in leukaemia survivorship. Central domains (i.e. coping and adjustment) are more intrapersonal, while external domains (i.e. healthcare system, HCPs, and community support) are more interpersonal. The red arrows indicate the tendency for participants to transition through more impactful (acute) and more enduring (chronic) seasons of survivorship

We identified six themes and two overarching themes. Together, these are represented in the Holistic Model of Leukaemia Survivorship (see Fig. 2).

### Theme 1: leukaemia is impactful, life-altering, and unexpected

Almost all participants described leukaemia as acutely life-changing and challenging. For most, diagnosis was incidental, unexpected, engendering feelings of bodily betrayal. Several participants were initially misdiagnosed after presenting non-specific symptoms (i.e. fatigue, shortness of breath, bruising, and bleeding).

I guess the word is bewildered. If I had to sum it up in one word, it’s just completely bewildered and just stripped of the things that I loved … [P15]

Treatment regimens differed, with some participants requiring extended inpatient care, and others outpatient treatment only. For most, high-intensity treatment started immediately after diagnosis, whereas some had to “watch and wait” for an extended period. Participants recounted diverse physiological impacts of initial treatment, describing surprise at their fragility. Most described their responsiveness to treatment as unpredictable, requiring specialist revision of treatment plans to “balance” treatment goals against life-threatening side-effects. While many participants conveyed mental resilience, most expressed feelings of shock, confusion, fear, sadness, and anger, describing the psychological toll as “horrendous” and difficult to process.

It’s horrible. It is bloody horrible and nobody can help you. … That’s why I’m really anti the lack of mental health preparation. [P123]

Most experienced fatigue, brain fog, and feeling physically unwell. Several reported issues of reduced bone density, peripheral neuropathy, infertility, medically induced menopause, and sexual dysfunction. Most participants (63%) who received a bone marrow or stem cell transplant developed graft versus host disease (GvHD) leading to a spectrum of secondary side-effects, including little-to-no natural immunity.

Stem cell transplant is a bloody awful physical experience to your body. I can't actually imagine many things worse, to be honest. [P61]

### Theme 2: leukaemia is enduring, limiting, and uncertain

Most participants discussed the chronic and demanding burden of leukaemia. Many described their treatment as “poison” and would “worry all the time” about the unknown and looming long-term health outcomes engendered by intense treatment regimens. This often required ongoing management of comorbid illnesses, immunodeficiency, and debilitating late-effects with limited health care practitioner (HCP) oversight.

I’ve had a lot of chemo and a lot of radiation ... All that can’t be good for you. [laughs]. For … all your organs and stuff. [P57] 

Several expressed frustration at having their late-effects neglected and reported feeling confused about which HCP to consult on specific issues. Despite taking comfort in positive monitoring outcomes, many described the “rigmarole” and emotional “rollercoaster” of long-term monitoring and centring their lives around the healthcare system. Some participants with chronic diagnoses described ongoing treatment as having “limitations” (i.e. medication timing) but considered it “a small price to pay to have the illness under control” [P19].

… the scary thing is there’s this stuff we can monitor for and can see coming and then there’s stuff that may just come out of nowhere. [P23]

This [cancer] is a worry and it’s a pain in the arse… getting follow-up treatment and check-ups. [P123]

While all participants valued remission and reassurance of 5-year survival, some felt in “limbo” and expressed concern the disease would mutate and/or progressively exhaust treatment options. Several took comfort in understanding the details and mechanisms of their treatment and diagnosis. Despite this, almost all participants discussed a heightened degree of self-monitoring and expressed difficulty differentiating between disease symptoms, side-effects, natural ageing, or other illnesses.

There’s still things that can go wrong. The drugs can lose their effectiveness…the leukaemia can find a way to mutate around it. [P19]

### Theme 3: survivorship is a team effort

A culture of advocacy was described as central to promoting quality support and meeting patient-specific needs. All participants discussed the importance of community, the healthcare system, and HCP support in facilitating adaptive navigation of leukaemia survivorship and reducing psychological strain. While some discussed heightened and damaging relational and familial strain introduced by their illness, many described life partners as their “chief carer”, “rock”, and “hero”, claiming their illness as “much harder on your carer than it is on you”. Several participants described their supporters as providing an “emotional bolster” and helping them feel “safe” and “wrapped-up” during treatment.

It makes you realise there really is some beautiful people out in the world. People love to help you. Wanna help you. [P57]

Many described leukaemia as a “steep learning curve” and had difficulty absorbing and processing medical information provided during diagnosis and active treatment. Conversely, participants who maintained personal records and involved a support person in consultations described meaningful benefits in filtering and comprehending medical information and decision-making. Some discussed not taking “no for an answer” and exercising agency and self-advocacy to ensure tailored care was received.

If you’ve got a doctor, he’s not telling you everything, not explaining everything, then you need a new doctor.”[P64]

... at the end of the day, you’re responsible for your own health. [P94]

“this was my life! And I had to get a grip on it. I wasn’t going to be just a… compliant person [P83]

Almost all participants indicated clear, collaborative, and proactive treatment planning demystified medical procedures and minimised anxiety. Most highlighted the importance of tailored, honest, and sensitive information sharing, valuing specialists who “…respected my boundaries in terms of what I did and didn’t want to know …”. However, several participants described stress and concern about specialists who went “through the motions”, neglected preparatory treatment information, and over-shared the “gory details”.

Many described the role of healthcare systems in facilitating positive inpatient care and support, including outpatient rehabilitation services. However, several felt like a “piece of meat”, having to “fight to get information” and mental health support during both inpatient and outpatient care. Others described losing “forward momentum” and connection with healthcare support when treatment ended.

It sort of feels in a way that the system kind of chews you up and spits you out when you’re having to re-acclimate to life after cancer. [P67]

### Theme 4: centrality of work as identity, focus, and financial security

Of the 17 participants working prior to diagnosis, 12 gradually returned to part- or full-time work. Despite gradual return to work, physical unpredictability due to fatigue, cognitive impairment, immunodeficiency, and GvHD limited work capacity and accelerated retirement for older participants. This was articulated as a loss of identity and provoked feelings of grief, jealousy, and bitterness. Nonetheless, most older participants described retirement as “really busy” and facilitated social contributions via community events.

The going-back-to-work thing is quite scary. You know, people say, ‘Oh, you know your body’. Well, here’s the thing, you don’t … [P15]

However, many participants of working age described feeling like “just a number”, unsupported and replaceable with enforced pause of studies or truncated careers. Several discussed the benefit of pre-existing workplace rapport and working from home flexibility in accommodating disease management and facilitating return to work. For almost all who wanted to work, regaining the ability to work or study was a “frustratingly slow” process associated with rebuilding their confidence and maintaining a “normal life”. Several participants found returning to work provided “purpose” and “meaning”, proving to themselves they were capable of “daily functioning”.

That was all part of me getting control back from the disease, and step one was back to work. [P126]

For some, significant financial toxicity arose from ongoing medical costs, rehabilitation programmes, and reduced income. This exacerbated the tension between disease management and premature return to work. Several discussed mitigating financial strain through income protection, medical insurance, a working partner, living with extended family, and flexible work arrangements.

When you’re an inpatient it’s all free. But when you become an outpatient, that’s when you start paying for it. I probably had a medication bill you couldn’t jump over. [P57]

… it costs to be unwell… it’s not just about not having the income… [P91]

### Theme 5: the dynamic landscape of coping

The cumulative uncertainty generated by changes in disease impact, late-effects, and survivorship contexts forced participants to regularly re-appraise their situation. This meant they relied on different coping strategies at different times across survivorship. Not all participants benefitted from strategies equally, but most routinely engaged with meaning-making (i.e. making sense of life events), problem-focused (i.e. directly addressing the issue), and emotion-focused (i.e. addressing emotions surrounding the issue) coping mechanisms. These coping strategies are detailed in Table 2.

It’s like a lot of things in life, there is no magic bullet. I’m not going to say something is going to suddenly make me feel wonderful. [P94]

**Table 2:** Coping strategies used by participants

Strategy	Examples	Illustrative quotes
Coping by cognitive reappraisal	*Positive reframing and acceptance* Majority fostered a positive disposition to “make the most” of their situation	“This sounds weird, but life’s been good. I don’t know. … It’s really good to be alive.” [P15]
	*Serenity and suppression* Participants prioritised controllable health-related factors to feel empowered	“Focusing on what I can control and realising there are things out of my control …” [P23]
	*Social comparison* Viewing themselves as “the lucky ones” relative to other survivors	“There’s always someone out there doing much worse than you’re doing, you know?” [P64]
	*Verbal processing* Processing experiences helped participants feel validated and understood	“It was just to talk to someone and just understand that, … this wasn’t a weakness or anything.” [P94] “A problem shared is a problem halved.” [P91]
Practical coping	*Exercise* Majority exercised to engender positive mental health and to escape rumination	“So for me … exercise things was about looking forward and leaving my disease for a little while, even for a minute…” [P15]
	*Recreation and goal setting* Many distracted themselves by exploring “joyful things” and keeping busy	“I’ve always … wanted to learn how to throw pots … that … would be good to process this sense of, ‘I’m in a new season’.” [P56]
Coping with others	*Quality time and travel* Togetherness with family and friends provided comfort	“We’re [family] just wanting to just do as many things as possible together and spend as much quality time as possible…” [P61]
	*Community support groups* Many participants explored shared experiences, helping them feel validated	“People spoke about … their journey and that … gave you a bit of reassurance that you weren’t doing it on your own.” [P112]
	*Coping with psychologists* Few participants gained effective coping techniques, while others felt predominantly misunderstood	“Social workers and the psychologists … was just a waste of time. They had no idea what they were dealing with.” [P123]
Religious coping	*Faith and spirituality* Some felt comforted by religion and used it as a “framework” to “process” leukaemia	“I think that [faith] has given me an anchor and … a purpose for living if you like.” [P19]

### Theme 6: survivorship as adjustment

All participants described adapting to physical and psychological changes, including FCR. Adjustment began at diagnosis but continued across survivorship, with individual supportive care needs varying depending on the severity of health outcomes. Perception of leukaemia as a transformative event was evident, with some participants reflecting a change in their appreciation of life.

I’ve been to a dark place a few times… there’s a combination of the diagnosis itself and getting your head around that... then there’s the managing of the side-effects and the impact they have on your life. [P137]

Adjusting to the psychological challenges and physiological late-effects perpetuated by treatment was central to navigating life with leukaemia. Participants recounted internally reconciling with new physiological restrictions and establishing more structured lifestyles to accommodate fatigue, immunodeficiency, GvHD, sexual dysfunction, and compromised mobility. Many described attempting to regain their old “normal” but found the persistent life-altering impact of leukaemia warranted ongoing rediscovery. Adjustment processes employed by participants are detailed in Table 3.

I did think my recovery would be more of an … upward trajectory. But it’s not. It’s the Swiss Alps…. it’s up and down and up and down, mountains. [P15]

**Table 3:** Processes of adjustment, disease impact, and recovery strategies

Adjustment	Example of impact	Example of recovery	Illustrative quote
Physical	*Fatigue* Majority felt debilitated by fatigue and unable to expend energy without “repercussions”	Many discussed accepting physiological limitations, not “fighting” themselves, and allowing rest	“… a lot of the time it’s walking the line … between pushing through … or letting my body take a break.” [P67]
	*Mobility* Several participants lost significant bone density, engendering “vulnerability”, and precluding exercise	Some benefitted from gradually increasing physical activity and “re-defining” their capacity	“… I’m in the process of adapting with my new disability, … I finally feel like I’m … re-adapting to normal life.” [P29]
	*Brain fog* Many compared brain fog to “cognitive decline” and described fear, distress, and “slower” cognitions	Some benefitted from cognitively stimulating activities, but several report no improvement across survivorship	“I struggle with the most basic things like how to get it out of Netflix and back into TV …and I’m mad with myself.” [P56]
	*Infertility* Younger participants described “really struggling” with the inability to plan or begin their own families	Several found comfort in understanding the benefits of treatment and exploring alternative options	“What else you would expect? … the other option is to die. … it’s not very helpful to have eggs if you are not alive.” [P61]
	*Sexual dysfunction* Some described the loss of “intimacy” and “identity” as “frustrating” and felt “stuck in a rut”	Few benefitted from medical interventions, but several felt reassured by understanding partners	“I have a very loving and accepting partner and we are able to talk about that stuff. So that makes it okay.” [P15]
Psychological	*Unpredictability of adjustment* Several described adjustment as “the Swiss Alps”, “feeling like somebody else”, “frustratingly slow”, and mentally taxing	Participants described taking “baby steps” and viewing adjustment as a process of “coming back together”	“… your body … has been through so much … poison … that everything takes time … it’s still not back to where I’d like it to be.” [P67]
	*Changing perspectives* Survival was viewed as a “privilege” and “leveller” where “every day was a bonus” bringing about changed values and priorities	Re-prioritised values often emphasised health and relationships over career and finance	“… I only have energy for those things that matter. And so it’s made me be really true to myself. And that’s privilege.” [P15]
	*Stress* Some participants became more sensitive to stress and experienced aversive physiological reactions	Several benefitted from avoiding potentially stressful situations and interactions	“… stress can be a cause of cancer. And I’m not happy with … being stressed. So I’m going for stress-free life.” [P64]
Fear of cancer recurrence	*Uncertain prognosis* Many described FCR as a “cancer cloud” that is “hanging over your head”, and a “visceral fear” that is difficult to process	Participants were comforted by “block[ing] it out”, the 5-year remission goal, consistent monitoring, and diligent haematologists	“…nobody really has an answer on this [FCR], and there isn’t one…who can tell you how long a piece of string is?” [P56]
	*Planning life around uncertainty* Participants described significant and enduring worry about the future and had difficulty making long-term plans	Several described “thriving… not just surviving”, “looking for … hope”, and found resilience in focusing on “whatever is coming tomorrow”	“I don’t know what the future holds, but I need to … be moving forward. I can’t afford to … go into a slump.… I don’t deserve it. So I’m not going to allow it.” [P57] “Well, me personally, then, I’ve accepted my journey. So my future is as explained in terms of my treatment plan.” [P137]

### Overarching theme 1: leukaemia survivorship is holistic

Common across all participant experiences were patient-centred domains (i.e. support networks, work and finance, coping mechanisms, and adjustment strategies; themes 3–6) and medical stages (i.e. diagnosis, treatment, and management and monitoring; themes 1–2) of leukaemia survivorship. Participants often described domains and stages as mutually influential and synchronous, suggesting the usefulness of domain-specific care changed according to the survivor, their medical stage, and leukaemia subtype (see Table 4 for comparison). Navigating these domains and stages necessitated ongoing re-evaluation of care needs and often led to heightened stress and uncertainty.

You’ve still got leukaemia symptoms, and they can be quite severe... Your world is tipped upside down... excoriated by all these different experiences that are new to you and your brain is trying to cope... you’ve got to acknowledge those feelings, the stress, anxiety, the unknown. You don’t know where this is going, you’re depressed, you’re angry... sometimes we [partner] would be sobbing... knowing that... next week all this could be over [death from disease]. [P83]

**Table 4:** Comparison of acute and chronic subtypes of leukaemia

	Acute subtype	Chronic subtype
*Diagnosis and treatment commencement*	Rapid symptom progression, increased likelihood to seek medical diagnostic testing	Increased likelihood of non-symptomatic disease progression and incidental diagnosis
	Rapid treatment commencement, typically inpatient	Typically watch and wait, monitored commencement. Both inpatient and outpatient treatment
	Immediate treatment commencement hinders health literacy preparation and HCP rapport-building	Pre-treatment delay provides time to improve health literacy and facilitate HCP shared decision-making
	Varies depending on risk stratification, and time of identification	Varies depending on risk stratification, and time of identification
*Treatment*	Requires curative treatment. Increased likelihood for SCT/BMT (44% of acute participants). Minimal ongoing treatment (18.75% of acute participants)	Typically require long-term management (63% of chronic participants). No SCT/BMT chronic participants
	If initial treatment unsuccessful in attaining remission	If initial treatment unsuccessful in attaining remission
*Monitoring and management*	Managing late-effects and comorbidities. Typically reported greater number extreme treatment-related of late-effects	Managing ongoing treatment side-effects (i.e. fatigue, cognitive impairment)

*SCT =* stem cell transplant, *BMT =* bone marrow transplant, HCP = Healthcare Professional

### Overarching theme 2: leukaemia survivorship is recursive

Participants described survivorship as non-linear and recursive, meaning survivors went through periods of *re-diagnosis* characterised by updated prognosis, diagnosis of late-effects, and re-evaluation of treatment regimens and management strategies. This was often due to treatment-related late-effects, rather than leukaemia-specific symptoms or relapse.

I thought I’d get to a point where I would barely have any hospital appointments... But that didn’t really happen because I did get complications and I still have to go to the hospital quite a few times a month... my expectations are definitely quite different from reality. [P29]

Consequently, all participants transitioned through periods of acute (i.e. impactful) and chronic (i.e. enduring) seasons of survivorship, binding them to the healthcare system.

I very naively thought I’d kind of have my treatment and then be okay. And then I thought I’d have my bone marrow transplant and I’d be okay. I wasn’t prepared for the length of the journey. [P15]

## Discussion

We qualitatively explored experiences of leukaemia survivors to determine whether the COSA Model of Survivorship Care (2016) (subsequently “the Model”) represents patient experiences. Our findings corroborate prior literature, emphasising the acute transition into survivorship upon diagnosis, ongoing need for follow-up care, and difficulty navigating the biopsychosocial impacts of leukaemia [15, 16]. We generated several novel findings, expanding current understanding of leukaemia survivorship. First, unlike prior research, while acute and chronic leukaemia subtypes have different impacts across survivorship domains (i.e. support networks, work and finance, coping mechanisms, and adjustment strategies), these domains are common across leukaemia holistically. Second, leukaemia survivorship appears to be a cyclical, transitioning through *acute* (i.e. impactful) and *chronic* (i.e. enduring) stages of survivorship, not a linear trajectory. Third, this process elicits varied physical and psychological impacts across all stages of survivorship, complicating long-term adjustment and requiring a range of coping strategies.

### Evaluating the COSA Model

The key domains of leukaemia survivorship we identified broadly map to the Model. In line with the Model, leukaemia survivorship is delineated into recursive stages of diagnosis, treatment, and monitoring and management (i.e. emergence of new problems). The Model meaningfully integrates and accounts for acute and chronic impacts of cancer, which we identified. While the Model *advises* tailored follow-up care based on patient-specific risk-stratification and needs assessment, participants often did not experience this type of survivorship care. Despite being broadly representative, the Model lacks leukaemia-specific characteristics detailing predominantly acute diagnosis requiring rapid commencement of treatment, which exacerbated anticipatory psychological distress, and limited disease and treatment education.

Corroborating prior literature, participant informational needs were predominantly unmet and information processing capacities differed [3, 32]. Poorer health literacy was compounded by intensive treatment regimens, aversive side-effects, and fragmented care, impeding participants’ ability to stay informed [32, 33]. Including active treatment as a critical stage in models, coupled with ongoing needs assessment to determine the appropriate recipient (e.g. survivor and/or primary caregiver), quantity, and tailoring of health information, may mitigate this barrier.

Contrary to the Model and recommendations for personalised risk-assessment and risk-stratification [34], while acute physical side-effects were commonly addressed at treatment completion, end of treatment was often sudden and few participants received personalised follow-up care plans or treatment summaries [35]. Chronic impact (i.e. late-effects) of leukaemia became evident over time, and the risks were not discussed at primary treatment completion. Substantiating prior findings**,** that as access to support from the cancer team diminished, rapid identification of new problems increased and access to ongoing care decreased [33, 36]. Personalised risk-stratification with transition plans and *ongoing* HCP late-effect consultations offer a feasible enhancement to follow-up care [37, 38].

### A holistic representation of leukaemia survivorship

While the Model represents the *ideal* components of follow-up care, implementation requires a patient-centred representation of leukaemia survivorship. As a broad all-cancer model, the Model does not entirely capture an understanding of leukaemia survivorship, particularly its interconnected and dynamic landscape. Extending the Model, we identified key patient-centred domains and stages of leukaemia survivorship and represent them in our Holistic Model of Leukaemia Survivorship (HMLS).

Our results suggest acute and chronic leukaemia have different implications across survivorship stages. The key differences originate from variability in treatment intensity and delivery. Acute leukaemia participants described more acute reactions to initial treatments and often had longer periods of inpatient care. The differential impacts of subtypes on key stages of leukaemia survivorship can be integrated into the HMLS. Further research investigating the implications of chronic relative to acute leukaemia survivorship across a larger sample will ensure generalisability. This may further specify subtype-specific survivorship interventions and implications.

### Strengths and limitations

The strength of this study is embedded in the diversity of participant experiences generated from Australia-wide recruitment, focused on survivors of leukaemia as a distinct category of haematological cancer.

Our sample primarily comprised married, highly educated, English-speaking participants accessing Australian healthcare. Therefore, theoretical transferability may differ across countries, cultures, and contexts depending on underlying healthcare services, level of patient education, and availability of community support.

The heterogenous collection of subtypes is a notable limitation. While our information power was enough to identify differences between acute and chronic subtypes, it did not allow us to establish a comprehensive mapping of those differences across survivorship. Inconsistencies between patient-reported survey and interview data precluded detailed quantification of treatment toxicities and other chronic health conditions. This further obscured valuable insight into the heterogeneity between leukaemia subtypes. Future research should further investigate the differential impacts of acute, chronic, and other leukaemia diagnoses across key survivorship domains and stages.

### Clinical implication and future direction

Our findings provide insight into the experiences of leukaemia survivorship and the factors influencing quality of care. Together, these findings establish the basis to operationalise and expand the COSA Model (2016) to capture the diverse experiences of leukaemia survivorship. Operationalisation of the Model needs to consider both logistical feasibility and intervention efficacy. Through implementing the HMLS as a supplementary framework to guide the Model, future research can (1) evaluate the Model efficacy across key domains and stages of leukaemia, (2) assess feasibility of implementing the Model across Australian healthcare systems, (3) explore and establish a more in-depth conceptualisation of primary leukaemia subtype-specific survivorship needs, and (4) investigate the mechanisms of interaction and causal relationships between survivorship domains.

Our research strongly suggested that high-quality advocacy stemming from patients, their community, healthcare systems, and HCPs is fundamental in facilitating survivorship care. The cornerstone of advocacy is information [39]. Therefore, we recommend patients and carers be provided tailored diagnostic and treatment information, including referrals to psychological and community-based support services, leukaemia-specific organisations, and administrative (i.e. legal, financial, employment, and insurance) support services. Future research could benefit from adapting, testing, and supplementing pre-existing psycho-oncology interventions [40] with the HMLS to guide and inform tailoring of therapy to the unique needs of leukaemia.

## Conclusion

We established a holistic representation of leukaemia survivorship, contributing valuable insight into the complex, impactful, and life-altering reality of living with leukaemia. We identified primary recursive domains and stages central to patient experiences across survivorship and conceptualised them in the HMLS. The COSA Model (2016) is theoretically consistent with our findings and offers a promising avenue for empirical validation and future development of holistic leukaemia-specific survivorship interventions. These novel findings may contribute to development of practical interventions and effective guidelines for HCPs to provide greater support for people living with leukaemia.

## Supplementary information

Below is the link to the electronic supplementary material.

Supplementary file1 (DOCX 26 KB)

Supplementary file2 (DOCX 17 KB)

Supplementary file3 (DOCX 311 KB)

## Data Availability

No datasets were generated or analysed during the current study.
