# Supplementary material for: The holistic model of leukaemia survivorship care: derived from a qualitative exploration of leukaemia survivorship
Source: Support Care Cancer. 2025 Mar 28;33(4):327. doi: 10.1007/s00520-025-09382-0 (PMC11953203; doi:10.1007/s00520-025-09382-0)
Supplement: Supplementary file 1 — Supplementary file1 (DOCX 26 KB) [file 520_2025_9382_MOESM1_ESM.docx]

**The Holistic Model of Leukaemia Survivorship Care: derived from a qualitative exploration of leukaemia survivorship**

Kirsten S^1^, Laidsaar-Powell R^1^, Shaw JM^1^, Dhillon, HM^1^.

Journal Name: Journal of Cancer Survivorship

Affiliations

1. Psycho-Oncology Cooperative Research Group, School of Psychology, Faculty of Science, The University of Sydney, NSW, Australia

ORCID IDs:

Kirsten S 0009-0000-5733-2055

Laidsaar-Powell R [0000-0002-3462-5645](https://orcid.org/0000-0002-3462-5645)

Shaw JM 0000-0002-9543-7066

Dhillon HM 0000-0003-4039-5169

Corresponding author: Haryana Dhillon,

Psycho-Oncology Cooperative Research Group,

School of Psychology, Faculty of Science,

The University of Sydney NSW 2006, Australia

[Haryana.dhillon@sydney.edu.au](mailto:Haryana.dhillon@sydney.edu.au)

**Supplementary File 1: Interview Schedule**

The initial interview schedule was pilot tested with a student-researcher and reviewed by a supervisor. The interview schedule was iteratively revised across data collection to maximise question relevance.

Initial Semi-Structured Interview Schedule

**Introduction and Consent**

Hello, my name is *[name of interviewer]* from *[institution]* calling about the experiences of people living with blood cancer study, is this still a good time to talk?

***If yes:*** Thank you for taking part in this study, I really appreciate your time. Before we begin the interview, I wanted to clarify a few things.

- The interview will approximately 1 hour. If you need to take a break, please let me know. We can complete this in two different sessions if you prefer. If you would like to stop this interview at any point, please let me know.
- The interview is being recorded so that I can concentrate on your responses and so I don’t have to take so many notes. Is that ok?
- I want to reassure you that your responses are completely confidential. It is ok to talk about positive or negative experiences that you had with your family or health professionals or workplace. Your family member, friends, health professionals, or workmates will not find out anything that has been said in this interview. Any papers or reports you will be deidentified.
- We will spend the first few minutes talking about your treatment. Then, I’d like to spend the bulk of our time hearing about your feelings and experiences since you finished your cancer treatment up until now.
- In this interview we will focus a lot on how life has been since you completed your main blood cancer treatment. By that, we mean your primary chemotherapy, radiotherapy, or transplant. That doesn't count ongoing targeted treatments like immunotherapy.
- Are you still willing to go ahead with this interview?
- Do you have any questions before we begin?

**Diagnosis and Treatment**

Can you tell me about your blood cancer diagnosis and where you are up to now with regards to treatment and monitoring?

Can you tell me about what it was like during your active treatment of blood cancer?

- What treatments did you receive?
- Is your treatment finished? Can you tell me a little bit about what your ongoing care involves?
- Have you been on, or offered, any immunotherapy (e.g. …. ) or targeted treatment (e.g…..) ? If so, can you tell me about your experience?

**Experience of Living with/beyond Blood Cancer**

Can you tell me what life has been like since completing your main cancer treatment, such as any ongoing impacts it has had on your day to day life?

How do you think your life has changed from before your leukaemia diagnosis?

**Living with blood cancer challenges and coping strategies/support**

Thinking about life now, what do you think have been the main challenges you have faced with regards to living with / beyond [blood cancer]?

- Do you feel like you live with any ongoing effects of the cancer / treatment? Can you tell me more about these, and how they impact your life?

*[This question will only be asked to certain participants who may be of the typical family planning age, <50 years old, as informed by the evidence base]*

Did your cancer / treatment impact on your family plans / fertility?

- If so, how? How do you feel about this?

*[Whilst some people might find this uncomfortable to discuss, it’s an important topic to explore. But feel free to skip this question if you prefer]*

Has the leukaemia / treatment impacted on your sexual life in any way?

- If so, how? How has this impacted on you?
- Any other things you want to mention?

You mentioned your experience *(side effects/symptoms/challenges/emotions)*, what helps you to get through that?

- What strategies do you use to help manage these?
- Have you received any help to manage these issues?

**Psychological Experiences/Coping**

Do you ever have any worries about the cancer returning or getting worse? Can you tell me more about this?

***If yes***: What are the main things you are worried about if the cancer does return or get worse?

How much can you keep these thoughts at bay, or how much does it intrude on your day?

- How do these feelings impact upon you?
- With a life-threatening disease like leukaemia, how much does the fear or worry about death and dying impact on you?

Is there anything that helps you adapt to life with (and after) leukaemia? On the other hand, what have you found is not helpful?

Do you feel comfortable discussing these worries with your healthcare team? Why/Why not?

How do you feel about the future?

What gives your life meaning?

**Healthcare Services**

How have you found follow-up care since you finished treatment?

- What have been positives?
- Have there been any negatives or challenges?

Are there any healthcare supports/services that help you cope?

- Do you think other services are needed?

Do you have a regular GP? How are they involved in your supportive care?

**Work and family**

Could you tell me about how your diagnosis of leukaemia has affected your ability to work / or your career?

- Has this changed over time?

Can you tell me about how the cancer has impacted on your family?

- Has this changed over time?

How have your family/friends been involved in your care since you completed your cancer treatment?

Has your diagnosis of leukaemia impacted your relationships, if so, how?

**Additional Comments**

Is there anything else you would like to add?

The interview has now come to an end. Thank you again for your participation and interest in our research.

Final Semi-Structured Interview Schedule

**Introduction and Consent**

Hello, my name is *[name of interviewer]* from *[institution]* calling about the experiences of people living with blood cancer study, is this still a good time to talk?

***If yes:*** Thank you for taking part in this study, I really appreciate your time. Before we begin the interview, I wanted to clarify a few things.

- The interview will approximately 1 hour. If you need to take a break, please let me know. We can complete this in two different sessions if you prefer. If you would like to stop this interview at any point, please let me know.
- The interview is being recorded so that I can concentrate on your responses and so I don’t have to take so many notes. Is that ok?
- I want to reassure you that your responses are completely confidential. It is ok to talk about positive or negative experiences that you had with your family or health professionals or workplace. Your family member, friends, health professionals, or workmates will not find out anything that has been said in this interview. Any papers or reports you will be deidentified.
- We will spend the first few minutes talking about your treatment. Then, I’d like to spend the bulk of our time hearing about your feelings and experiences since you finished your cancer treatment up until now.
- In this interview we will focus a lot on how life has been since you completed your main blood cancer treatment. By that, we mean your primary chemotherapy, radiotherapy, or transplant. That doesn't count ongoing targeted treatments like immunotherapy.
- Are you still willing to go ahead with this interview?
- Do you have any questions before we begin?

**Diagnosis and Treatment**

Can you tell me about your blood cancer diagnosis and where you are up to now with regards to treatment and monitoring?

Can you tell me about what it was like during your active treatment of blood cancer?

- How was your interaction with your HCP during this time?
- Can you tell me about your transition out of active/initial treatment?
- Have you been on, or offered, any immunotherapy (e.g. …. ) or targeted treatment (e.g,…) ? If so, can you tell me about your experience?

**Healthcare Services**

How have you found follow-up care since you finished treatment?

- Have there been any negatives or challenges?

Are there any healthcare supports/services that help you cope?

Do you have a regular GP? Are they involved in your supportive care?

**Experience of Living with/beyond Blood Cancer**

Can you tell me what life has been like since completing your main cancer treatment, such as any ongoing impacts it has had on your day to day life?

Has your life has changed from before your leukaemia diagnosis?

**Living with blood cancer challenges and coping strategies/support**

Thinking about life now, what do you think have been the main challenges you have faced with regards to living with / beyond [blood cancer]?

- Do you feel like you live with any ongoing effects of the cancer / treatment? Can you tell me more about these, and how they impact your life?

*[The following question will only be asked to certain participants who may be of the typical family planning age, <50 years old, as informed by the evidence base]*

Did your cancer / treatment impact on your family plans / fertility?

- If so, how? How do you feel about this?

*[Whilst some people might find this uncomfortable to discuss, it’s an important topic to explore.]*

Has the leukaemia / treatment impacted on your sexual life in any way?

- If so, how?
- Has this impacted on you and your relationships?

You mentioned your experience *(side effects/symptoms/challenges/emotions)*, what helps you to get through that?

- What strategies do you use to help manage these?
- Have you received any help to manage these issues?

**Psychological Experiences/Coping**

Do you ever have any worries about the cancer returning or getting worse? Can you tell me more about this?

***If yes***: What are the main things you are worried about if the cancer does return or get worse?

How much can you keep these thoughts at bay, or how much does it intrude on your day?

- Does the fear or worry about death and dying impact on you?

Have you found the need to make any life adjustments to accommodate your illness?

***If yes:*** Is there anything that helps you adapt to life with (and after) leukaemia? On the other hand, what have you found is not helpful?

Do you feel comfortable discussing these worries with your healthcare team? Why/Why not?

**Work and family**

Could you tell me about how your diagnosis of leukaemia has affected your ability to work / or your career?

- Has this changed over time?

How have your family/friends been involved in your care since you completed your cancer treatment?

- Has your diagnosis of leukaemia impacted on your family or friendships? How have you navigated this?

**Additional Comments**

Is there anything else that you think I might've missed or that you would like to add?

Thank you again for your participation and interest in our research.
