# Supplementary material for: The holistic model of leukaemia survivorship care: derived from a qualitative exploration of leukaemia survivorship
Source: Support Care Cancer. 2025 Mar 28;33(4):327. doi: 10.1007/s00520-025-09382-0 (PMC11953203; doi:10.1007/s00520-025-09382-0)
Supplement: Supplementary file 2 — Supplementary file2 (DOCX 17 KB) [file 520_2025_9382_MOESM2_ESM.docx]

**The Holistic Model of Leukaemia Survivorship Care: derived from a qualitative exploration of leukaemia survivorship**

Kirsten S^1^, Laidsaar-Powell R^1^, Shaw JM^1^, Dhillon, HM^1^.

Journal Name: Journal of Cancer Survivorship

Affiliations

1. Psycho-Oncology Cooperative Research Group, School of Psychology, Faculty of Science, The University of Sydney, NSW, Australia

ORCID IDs:

Kirsten S 0009-0000-5733-2055

Laidsaar-Powell R [0000-0002-3462-5645](https://orcid.org/0000-0002-3462-5645)

Shaw JM 0000-0002-9543-7066

Dhillon HM 0000-0003-4039-5169

Corresponding author: Haryana Dhillon,

Psycho-Oncology Cooperative Research Group,

School of Psychology, Faculty of Science,

The University of Sydney NSW 2006, Australia

[Haryana.dhillon@sydney.edu.au](mailto:Haryana.dhillon@sydney.edu.au)

**Supplementary File 2: Reflective Thematic Analysis Procedure**

This document describes the steps taken to analyse the interview data using Reflective Thematic Analysis.

*(1)* *Familiarising yourself with the data set*: The researcher conducted all but one of the interviews, familiarising themselves with the patient-reported outcome measures prior to each interview, noting their initial impressions, considerations, and biases before and after each interview. The researcher transcribed all but one of the interviews, checked, edited and de-identified the transcripts, and listened to all audio-recordings at least once. Each transcript was read multiple times during analysis.

*(2)* *Coding*: Generating and collating concise codes (labels), coupled with data extracts across the entire dataset, to capture key features relevant to the research question. Three transcripts (20%) were independently coded by both supervisor and primary researcher to encourage naïve identification of codes, ensure rigour, and challenge potential biases and misinterpretation of the data.

*(3)* *Generating initial themes*: Using the cross-coded transcripts, the researcher iteratively developed eight candidate themes consisting of thirty-eight subthemes. Subthemes were further partitioned into sub-subthemes to account for additional detail and develop a deeper understanding of the data.

*(4)* *Developing and reviewing themes*: Candidate themes and sub-themes were assessed for accuracy and iteratively updated, discarded, or combined as they were applied to the entire dataset. Transcripts and codes were iteratively reviewed to ensure appropriate interpretation and understanding of the data.

*(5)* *Refining, defining, and naming themes*: Informative names were developed as the scope of each theme and sub-theme was defined. This required detailed analysis and understanding of the data.

*(6)* *Writing up*: The data was presented as an analytical narrative detailing the relevant contribution of each theme as a shared experience across leukaemia survivorship.
