# Supplementary material for: The holistic model of leukaemia survivorship care: derived from a qualitative exploration of leukaemia survivorship
Source: Support Care Cancer. 2025 Mar 28;33(4):327. doi: 10.1007/s00520-025-09382-0 (PMC11953203; doi:10.1007/s00520-025-09382-0)
Supplement: Supplementary file 3 — Supplementary file3 (DOCX 311 KB) [file 520_2025_9382_MOESM3_ESM.docx]

**The Holistic Model of Leukaemia Survivorship Care: derived from a qualitative exploration of leukaemia survivorship**

Kirsten S^1^, Laidsaar-Powell R^1^, Shaw JM^1^, Dhillon, HM^1^.

Journal Name: Journal of Cancer Survivorship

Affiliations

1. Psycho-Oncology Cooperative Research Group, School of Psychology, Faculty of Science, The University of Sydney, NSW, Australia

ORCID IDs:

Kirsten S 0009-0000-5733-2055

Laidsaar-Powell R [0000-0002-3462-5645](https://orcid.org/0000-0002-3462-5645)

Shaw JM 0000-0002-9543-7066

Dhillon HM 0000-0003-4039-5169

Corresponding author: Haryana Dhillon,

Psycho-Oncology Cooperative Research Group,

School of Psychology, Faculty of Science,

The University of Sydney NSW 2006, Australia

[Haryana.dhillon@sydney.edu.au](mailto:Haryana.dhillon@sydney.edu.au)

**Supplementary File 3: Consolidated Criteria for Reporting Qualitative Research (COREQ)**

This document contains the completed COREQ checklist, which is used to assess the methodological rigour and transparency of qualitative research.

**Completed COREQ Checklist**


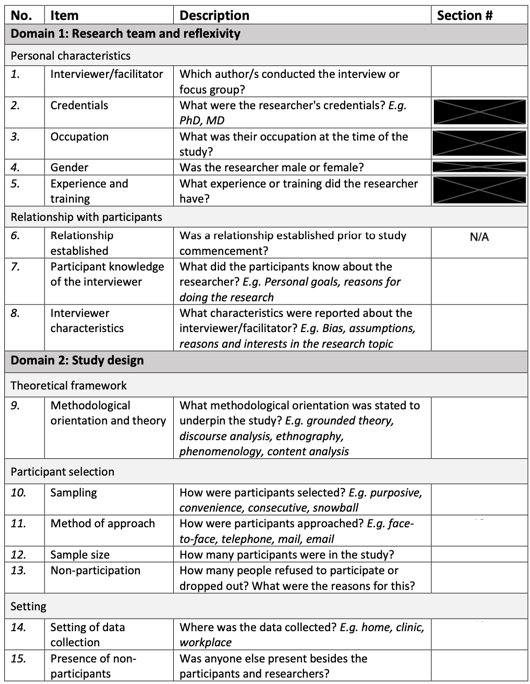


N/A

p. 8

p. 6-7

p. 6-7

p. 8

p. 6

p. 6

p. 6

N/A

p. 6

p. 19


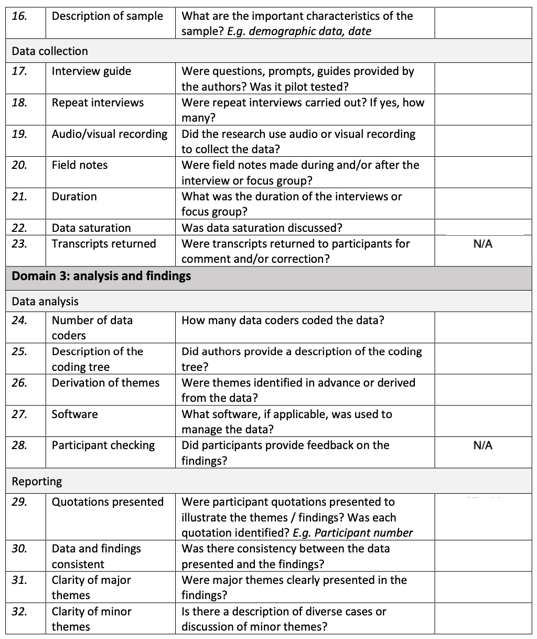


N/A

N/A

p. 8-15

p. 8-15

p. 8-15

p. 8-15

p. 6-7

p. 7

p. 20

p. 7, 19

p. 6

p. 8

N/A

p. 6

N/A

p. 7

p. 8
